# Temperature and Safety Profiles of Needle-Warming Techniques in Acupuncture and Moxibustion

**DOI:** 10.1155/2012/168393

**Published:** 2012-06-12

**Authors:** X. Y. Gao, C. Y. Chong, S. P. Zhang, K. W. E. Cheng, B. Zhu

**Affiliations:** ^1^School of Chinese Medicine, Hong Kong Baptist University, Hong Kong; ^2^Institute of Acupuncture and Moxibustion, China Academy of Chinese Medical Sciences, Beijing 100700, China; ^3^Department of Electrical Engineering, The Hong Kong Polytechnic University, Hong Kong

## Abstract

The needle-warming technique combines acupuncture and moxibustion, and it is commonly practised in China to relieve pain conditions. However, burning of moxa has many disadvantages. This study examined the temperature and safety profiles of such technique. First, skin temperature changes during needle-warming were examined in anesthetized animals to determine the safe distance for needle-warming moxibustion in human subjects. Then, the practical distance for needle-warming in human subjects were verified. Finally, the temperature profiles of the needle during needle-warming moxibustion were examined using an infrared camera. Our results show that during needle-warming moxibustion there is little heat being conducted into deep tissue via the shaft of the needle, and that the effective heating time to the acupoint is rather short compared to the period of moxibustion. These findings suggest that the needle-warming technique is an inefficient way of acupoint thermal stimulation and should be modified and improved using new technologies.

## 1. Introduction

Acupuncture and moxibustion (Zhenjiu), which is an ancient Chinese therapy that uses physical stimulation on body surface to alleviate diseases, is gaining popularity in many countries because it is free from side effects of chemical drugs [1–5]. Still, much remains unknown about the characteristics of the therapy, its efficacy, and the underlying mechanisms of actions, especially for moxibustion. A better understanding of the physical characteristics of the therapy is important for future research, as it will help to improve the reproducibility of the intervention, standardize the intervention through development of new devices, and determine the adequate controls in clinical trials [6, 7]. 

The needle-warming technique involves stimulating acupuncture points by needle penetration followed by burning of a piece of moxa attached to the needle. It is often used for treatment of pain conditions such as arthritis, sciatica, cervical spondylopathy, intervertebral disk herniation, and osteoarthritis [8–12]. It is generally believed that heat from burning of moxa (moxibustion) is transmitted to the acupoint by radiation as well as by direction conduction via the shaft of the needle, thereby stimulating deep tissue within the acupoint, besides warming the acupoint on the surface [13]. The physiological basis of such practice is not well understood. Nevertheless, it has been reported that moxibustion can lead to the release of anti-inflammatory agents, such as heat shock proteins [14]. However, burning of moxa has several disadvantages. In particular, smoke produced by combustion of moxa may trigger the onset of an asthmatic attack [15, 16]. It is also time consuming to apply the technique and difficult to control the heat to prevent burn injury to the skin [17–20]. Therefore, a better understanding of the thermal characteristics of the needle-warming technique can facilitate the development of new therapeutic devices and techniques that have the same characteristics of the technique without the drawbacks. To this end, we investigated the profiles of heat transmission during needle-warming moxibustion.

## 2. Material and Methods

Experimental procedures were approved by the committee on the use of human and animal subjects in teaching and research of Hong Kong Baptist University.

### 2.1. Measurements in Anesthetized Animals

Three rabbits weighing between 1.8 kg to 2.05 kg were used. They were anaesthetized with an intravenous injection of pentobarbitone (30 mg/kg) with supplementary doses added when necessary, and euthanized using over dose of pentobarbital at the end of the experiment. The skin around the acupoint Huan Tiao (GB30) was shaved to expose an area approximately 6 × 6 cm^2^, and the animal was placed on a thermostatic electric heating blanket to keep the body temperature at around 37.5 ± 0.5°C. The room temperature was kept at 23°C. A small temperature probe (MLT409/D ADInstruments) was placed on the skin surface immediately adjacent to the point of needle insertion. Signals from the temperature probes were processed by a data acquisition system (ADInstruments).

### 2.2. Measurements in Human Subjects

Six healthy volunteers were invited to participate in the experiment on separate days. The room temperature was maintained at 23°C. To measure skin temperature, a skin probe (ADInstruments, model MLT409/D; length × diameter: 2.8 × 9.6 mm) was placed immediately adjacent to the point of needle insertion, and the signal recorded from the probe was processed as described above.

### 2.3. Acupuncture and Moxibustion Procedures

Two different diameters (0.30 mm × 40 mm and 0.35 × 40 mm) of stainless steel acupuncture needles (Carbo, Suzhou, China) with a handle size of 1.0 mm × 33.6 mm were tested at acupoint Huan Tiao (GB30) in animal experiments and at acupoint Zusanli (ST36) in human subjects. A 15 mm long moxa cylinder block weighing 1.70 ± 0.05 g, which was cut from moxa sticks (Wu She Pure Moxa Roll, Suzhou, China), was attached to the handle of the needle by inserting the handle into the center of the block. The distance from the bottom of the moxa block to the junction of handle and shaft of the needle was approximately 10 mm. In a few experiments, commercially available moxa cylinders (12 mm × 15 mm; Wushe, Shuzhou, China) were tried, but were not studied further because their effective heating time on the skin surface was very brief. That is, the duration of skin temperature above 40°C during burning of these moxa cylinders was less than 30 seconds when the cylinder was at 10 mm above the skin. In some human experiments, a thin piece of cardboard (approximately 50 mm × 50 mm × 0.2 mm) was placed above the skin to mimic clinical practice procedures, which aimed to prevent possible burn injury caused by falling of moxa ash. The moxa was ignited at the top in each of the trial.

### 2.4. Measurement of Temperature on Acupuncture Needles

An acupuncture needles were painted black with a permanent marker (ZEBRA) and held vertically by a clamp at the end of the handle, with a moxa block attached to the handle and ignited from the top. To measure temperature of the acupuncture needle, a thermal tracer (NEC, model TH9100PMV) equipped with a close-up lens (TH-386) was used. The emissivity index of the thermal tracer was set to 0.99. The accuracy of the tracer measurement was checked by placing the needle in an enclosed thermostatic box, with the needle attached to the tip of an alcohol-filled thermometer (Hisamatsu, Japan). It was found that the difference between the thermometer reading and the tracer reading was less than 0.5°C in the range of 30–45°C, which was acceptable for the purpose of the current experiments. 

## 3. Results

### 3.1. Safe Distance between the Moxa Block and the Skin Surface in Needle-Warming Moxibustion

**Figure 1:**
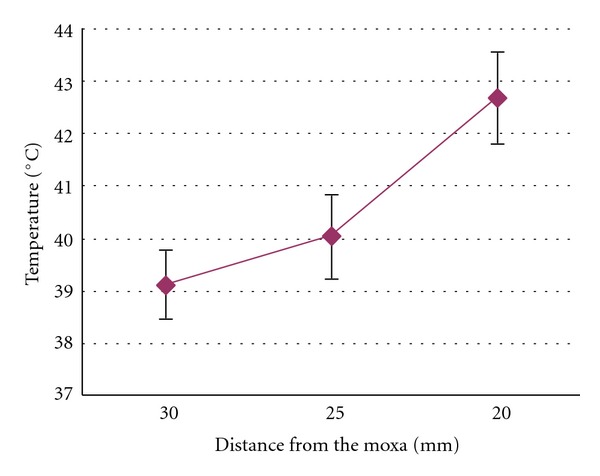
Line chart showing the relationship between the distance of moxibustion and the temperature on the skin surface of acupoint GB30 in anaesthetized rabbits during needle-warming (mean ± SD, *n* = 6). *X*-axis indicates the distance between the moxa block and skin surface.

We first investigated the safe distance between the moxa block and the skin surface in needle-warming moxibustion using anesthetized rabbits. With a moxa block (1.70 ± 0.05 g) attached to an inserted acupuncture needle, the maximum mean temperature (±SD) on the skin surface directly under the ignited moxa block was 39.1 ± 1.2°C, 40.0 ± 2.3°C, or 42.6 ± 2.0°C when the distance between the moxa block and the skin surface was 30 mm, 25 mm, or 20 mm, respectively (Figure 1). No significant difference was seen in the maximum mean temperature on the skin surface between two different diameters of needles. In one animal, the moxa block was placed at 15 mm above the skin, and the temperature of the skin surface exceeded 46°C, which induced a superficial burn, at which point the moxa block was quickly removed. These results indicated that for moxa block of this size, the safe distance between the moxa block and the skin surface must be over 20 mm, and burning the moxa block at a distance less than 20 mm above the skin would be unsafe in human subjects. 

### 3.2. Skin Temperature Changes during Needle-Warming in Human Subjects

**Figure 2:**
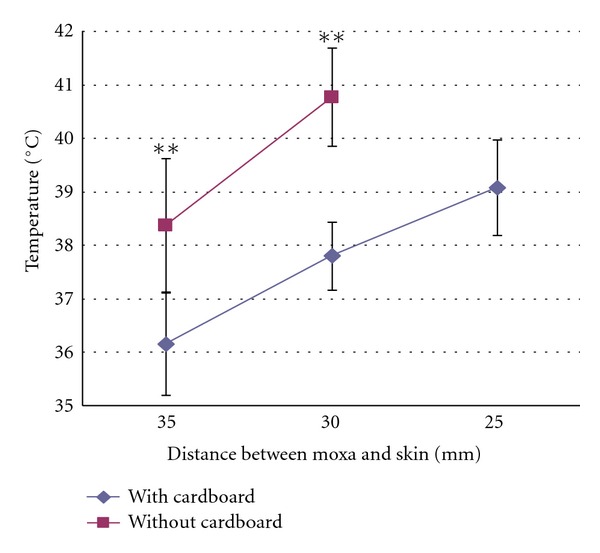
Line chart showing the relationship between the distance of moxibustion and the temperature on the skin surface of acupoint ST36 of human subjects during needle-warming (mean ± SD, *n* = 6). *X*-axis indicates the distance between the moxa block and skin surface. Measurements were taken either with cardboard overlying the acupoint (with cardboard) or without (without cardboard). **P < 0.01.

The temperature on the skin surface was studied in 6 human subjects, either with or without cardboard covering the acupuncture area. As seen in Figure 2, without cardboard covering the acupuncture area, the skin temperature was 38.4 ± 1.3°C and 40.8 ± 0.9°C when the distance between the moxa and the skin surface was 35 mm and 30 mm, respectively. The maximum skin temperature reached was 41.9°C at 30 mm distance in one occasion, at which point the volunteer reported intense heat sensation in the area surrounding the needle, but it was not painful. Moxibustion distance less than 30 mm without cardboard covering was not tested, as it might cause severe pain and even skin burn injuries. 

**Figure 3:**
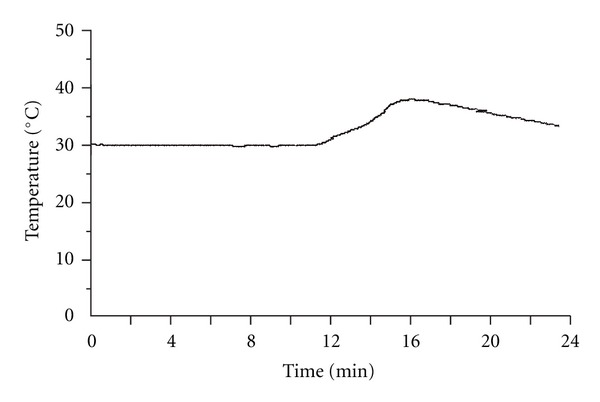
Computer chart record showing temperature changes during needle-warming in a human subject. The distance between the moxa block and the skin of acupoint ST36 was 35 mm. No cardboard cover was placed on the surface of the acupoint.

With cardboard covering the acupuncture point and the surrounding area, the skin temperature was 36.2 ± 1°C, 37.8 ± 0.6°C, and 39.1 ± 0.9°C, when the distance between the moxa and the skin surface was 35, 30, and 25 mm, respectively (Figure 3). 

For a given distance, the average maximum skin temperature reached in the group without cardboard cover was higher than that with cardboard cover, with the mean difference ranges from 1.2°C to 2.9°C (*P* < 0.01, *t* test).

The time course of the skin temperature change during needle-warming is illustrated in Figure 3. It can be seen that there was no increase in skin temperature after ignition of the moxa in the first 12 minutes also. Then, there was a gradual increase and a gradual decrease in temperature between 12–24 minutes. It was observed that the skin temperature did not increase significantly until the lower part of moxa started to burn, and the effective heating period (i.e., >37°C) lasted only 2-3 minutes.

### 3.3. Temperature Distribution along Needle Shaft during Needle-Warming Moxibustion

**Figure 4:**
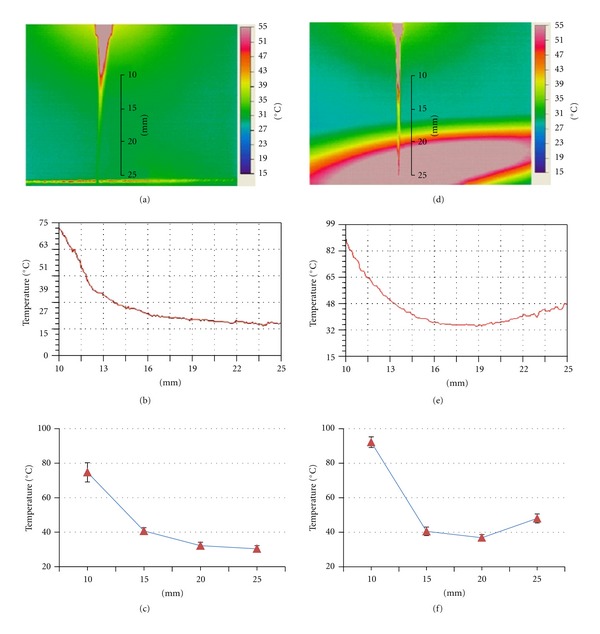
(a) and (d) thermographic images showing temperature along the needle during the hottest period of needle-warming. (b) and (e) temperature reading of the needles as shown in (a) and (d), respectively. The *X*-axis indicates the distance from the moxa block. (c) and (f) line graphs showing mean temperature reading of the needles at fix distances from the moxa block during the hottest period of needle-warming (*n* = 9 experiments). In (a), (b), and (c), a cotton thread was placed 25 mm below the moxa block perpendicularly near the needle to facilitate measurements. In (d), (e), and (f), a piece of cardboard was placed 25 mm below the moxa block with the needle piercing through it.

The temperature of the needle shaft was measured with the needle suspended in the air. As seen in Figures 4(a), 4(b), and 4(c), the temperature decreased exponentially along the shaft during needle-warming, dropping below 35°C at distance over 20 mm away from the burning moxa block. However, with a piece of cardboard placed 25 mm under the moxa block, mimicking the clinical situation in which the skin below the moxa block was covered, the temperature distribution along the needle shaft was bimodal (Figures 4(d), 4(e), and 4(f)). The lowest temperature point was found around 18 mm from the moxa block, where the maximal temperature reached was 35°C. After this point, the shaft temperature increased gradually towards the cardboard, reaching a maximum of 48°C at the juncture of the needle and the cardboard. 

## 4. Discussion

This is the first study that measures the temperature of acupuncture needle shaft during needle-warming technique using an infrared camera. We found that the effective heating distance of the needle shaft was limited to less than 20 mm from the moxibustion site. On the other hand, at least 25 mm was required between the moxibustion site and the skin surface for safe needle-warming practice. Thus, there is no effective direct heat transmission from the needle to the skin tissue and below in needle-warming. We also found that the overall heating time of the acupoint in needle-warming was rather brief, lasting only 2-3 minutes, comparing with the 25 minutes that were required to burn the whole block of moxa. The implications of these findings are discussed below.

It has been shown that temperature over 40°C is required to produce physiological effects, but thermal pain sensation occurs at 44.4 ± 2.1°C, with some variations between individual [21–26], and tissue injury occurs if temperature is maintained at over 45°C [27]. In other words, the temperature window for therapeutic heat treatment without causing pain is relatively narrow, between 40–42°C. Our study showed that in heat treatment with needle-warming technique, the skin temperature varied a lot, depending on the distance between the skin and the moxa block, as well as the size of the moxa block. For smaller moxa blocks, such as those commercially available ones that had been tested, the effective heating time might be too short to be effective. For larger moxa blocks, such as those used in the current study, the distance between the skin and the moxa block must be at least 25 mm even with cardboard protection or 30 mm without protection. 

It is interesting to find that at sites over 20 mm below the moxa block, before the needle touching the skin, the needle shaft temperature was below 40°C if no object was placed underneath. This needle temperature would have little therapeutic effect. On the other hand, if a piece of cardboard was placed 25 mm below the moxa block, after dropping below 40°C at about 20 mm below the moxa block the temperature of the needle shaft increased gradually again as it approached the cardboard underneath, and reached about 48°C at the contact point between the needle and the cardboard. The second increase in shaft temperature was presumably due to reflection of heat radiation by the cardboard underneath. However, cautions must be taken when interpreting the temperature reading of the tracer because we have not controlled for the effect of stray IR, which might came from any sources other than the needle. Two factors may influence the effect of stray IR on the reading of the tracer: (i) the ratio of the intensity of the stray IR to the true IR signal from the needle and (ii) the state of the stray IR. The stray IR in the current experiment is from stationary objects, and by using averaged measurements, the variation in stray IR had been minimized. However, because the temperature of the needle shaft 20 mm below the moxa block was below 40°C, and the needle shaft was very thin, the intensity of the signal might be very low. Thus, the stray IR might distort the reading of the tracer on the part of the needle near the cardboard, resulting in higher reading than the true temperature of the needle. Such influence has only a short distance and therefore only affected less than 4 mm of the needle near the cardboard. Nevertheless, such potential measurement error has no impact on the interpretation of the main findings. 

Until now, it is generally believed that heat from burning of moxa can be transmitted to the acupoint via the shaft of the needle, in addition to heat radiation [13]. Therefore, the needle-warming technique allows heat being conducted into deeper part of the acupoint hence resulting in thermal stimulation of deep tissue [28], whereas moxibustion with a moxa stick only has superficial effects on the skin [29]. Our findings suggest that at the current setting, which is similar to clinical practice, there is only radian heat stimulation to the acupuncture point, as heat from the needle is lost at a distance over 20 mm, before the needle penetrates the skin. This finding is consistent with the recent report by Cheng et al. [13], in which a maximum of 41.71 ± 1.39°C was reported on the contact point between the skin and the stainless steel needle.

## 5. Conclusion

The present findings indicate that the needle-warming practice is an inefficient heat stimulation by combustion of moxa because there is no direct heat transmission into the tissue via the acupuncture needle. For radian heat stimulation of the acupoint surface, other techniques of moxibustion, such as using a moxa box, may be safer and more efficient. New technologies, such as electrical heating devices, can be used in the future to improve the needle-warming technique. We also show that the heat conduction process can be visualized with an infrared camera, and such method will be useful in developing new instruments that can replace the needle-warming technique.
